# Injured Endothelial Cell: A Risk Factor for Pulmonary Fibrosis

**DOI:** 10.3390/ijms24108749

**Published:** 2023-05-14

**Authors:** Weiming Zhao, Lan Wang, Yaxuan Wang, Hongmei Yuan, Mengxia Zhao, Hui Lian, Shuaichen Ma, Kai Xu, Zhongzheng Li, Guoying Yu

**Affiliations:** State Key Laboratory of Cell Differentiation and Regulation, Henan International Joint Laboratory of Pulmonary Fibrosis, Henan Center for Outstanding Overseas Scientists of Organ Fibrosis, Institute of Biomedical Science, College of Life Science, Henan Normal University, Xinxiang 453007, China; zhaoweiming@stu.htu.edu.cn (W.Z.); wanglan@htu.edu.cn (L.W.); wangyaxuan@stu.htu.edu.cn (Y.W.); lianhui@stu.htu.edu.cn (H.L.); mashuaichen@stu.htu.edu.cn (S.M.); xukai@stu.htu.edu.cn (K.X.); lizhongzheng@stu.htu.edu.cn (Z.L.)

**Keywords:** pulmonary fibrosis, endothelial cells, endothelial–mesenchymal transition, myofibroblasts

## Abstract

The pathological features of pulmonary fibrosis (PF) are the abnormal activation and proliferation of myofibroblasts and the extraordinary deposition of the extracellular matrix (ECM). However, the pathogenesis of PF is still indistinct. In recent years, many researchers have realized that endothelial cells had a crucial role in the development of PF. Studies have demonstrated that about 16% of the fibroblasts in the lung tissue of fibrotic mice were derived from endothelial cells. Endothelial cells transdifferentiated into mesenchymal cells via the endothelial–mesenchymal transition (E(nd)MT), leading to the excessive proliferation of endothelial-derived mesenchymal cells and the accumulation of fibroblasts and ECM. This suggested that endothelial cells, a significant component of the vascular barrier, played an essential role in PF. Herein, this review discusses E(nd)MT and its contribution to the activation of other cells in PF, which could provide new ideas for further understanding the source and activation mechanism of fibroblasts and the pathogenesis of PF.

## 1. Introduction

Pulmonary fibrosis (PF) is the end-stage consequence of various interstitial lung diseases (ILD). It is a chronic progressive disease with an average survival of 3–5 years after diagnosis [1]. PF results from the dysregulation of alveolar epithelial cell (AECs) repair in response to alveolar and vascular damage, which leads to the excessive accumulation of ECM, proliferation of myofibroblasts, distortion of pulmonary architecture, and loss of pulmonary tissue function [2]. Studies have reported that circulating fibrocytes, pulmonary alveolar epithelial cells, fibroblasts, pericytes, macrophages, and endothelial cells were the progenitors of myofibroblasts and contributed to the development of PF.

Endothelial cells (ECs) comprise 30% of the lung cellular composition; it has been recognized that the loss of pulmonary microvascular endothelial cells (PMVECs) and deregulated angiogenesis are critical biological processes of pulmonary fibrosis [3,4]. Studies pointed out that ECs may be involved in pulmonary fibrosis through involving the production of the gaseous free radical nitric oxide or/and transforming into myofibroblasts during fibrogenesis [5]. During PF, ECs lost EC markers and functions and gained mesenchymal cell markers and characteristics through a process called E(nd)MT [6]. However, studies of ECs and their role in PF appear to have fallen behind other cell studies. We review the contribution of ECs to PF, which could provide new ideas for further understanding the role of ECs in PF and the pathogenesis of PF.

## 2. Pulmonary Fibrosis

More than 200 causes are known to induce PF, including genetic factors, autoimmune deficiency, environmental exposure, viral infection, etc. Pulmonary fibrosis caused by injuries can lead to different degrees of impaired gas exchange, inducing dyspnea, coughing, fatigue, and significant deterioration in quality of life, even resulting in death [7]. PF affects an increasing number of people and has become one of the fastest-growing global healthcare concerns.

### 2.1. Etiology of Common Pulmonary Fibrosis

**Figure 1:**
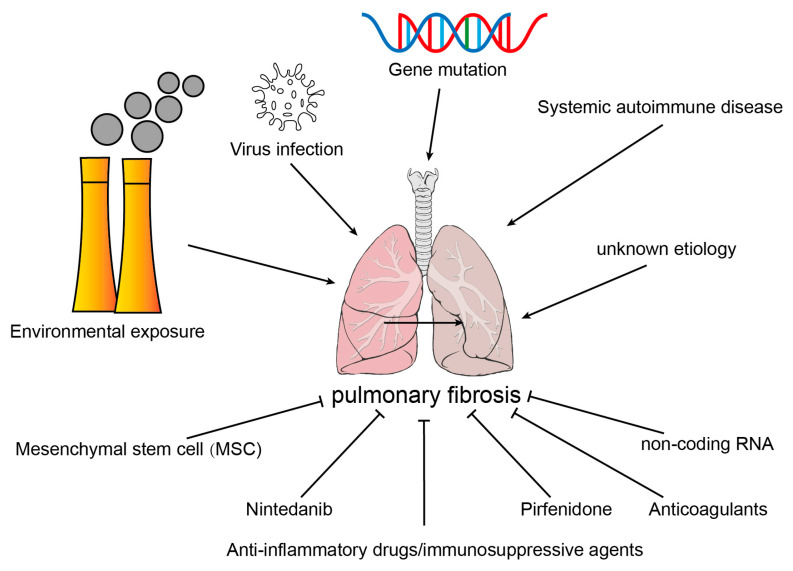
Etiology and therapeutic drugs of pulmonary fibrosis.

According to the etiology, PF was divided into secondary pulmonary fibrosis with known etiologies, PF caused by genetic mutation, and idiopathic pulmonary fibrosis (IPF) with unknown etiology. At present, anti-inflammatory drugs/immunosuppressants, anti-fibrosis drugs, anticoagulant drugs, mesenchymal stem cells, non-coding RNA, and lung transplantation have been used in treating pulmonary fibrosis; however, there is still no effective remedy for this disease [8] (Figure 1).

#### 2.1.1. Environmental Exposure

PM2.5 (particulate matter less than 2.5 μm in aerodynamic diameter), asbestos, silica, and fumes could induce the accumulation of reactive oxygen stress (ROS) in type II alveolar epithelial cells (AECII) of lung tissue, which leads to oxidative stress and inflammatory damage in lung tissue [9]. A study reported that PM2.5 instillation-induced lung inflammation and fibrosis was associated with aberrant epithelial–mesenchymal transition (EMT), ROS, mitochondrial injury, and mitophagy [10]. Compared with WT, AECII from Ogg1^−/−^ mice presented increased mtDNA damage and reduced mitochondrial aconitase expression, and these changes were intensified at 3 weeks after crocidolite exposure [11]. Environmental exposure-induced PF may be closely related to oxidative stress, mitochondrial damage, and mitophagy in epithelial cells.

#### 2.1.2. Virus Infection

Human immunodeficiency virus (HIV), cytomegalovirus (CMV), murine gamma-herpes virus 68 (MHV-68), avian influenza virus, and other viruses may result in PF [12]. Viruses may induce pulmonary fibrosis through two pathways. First, the virus induces persistent damage and abnormal damage repair of alveolar epithelial cells, leading to the development of PF. Second, lung injury induced inflammatory infiltration and the activation of the immune system. Immune cells aggregate in the injured lung tissue and release many proinflammatory and profibrotic factors, including TGF-β, TNF-α, MMPs, and interleukin. This induces sustained lung damage and PF [13].

#### 2.1.3. Systemic Autoimmune Disease

Systemic sclerosis (SSc), rheumatoid arthritis (RA), polymyositis/dermatomyositis (PM/DM), and Sjögren’s syndrome (SS) may promote inflammatory responses in lung inflammation through TGF-β, TGF-β-mediated pulmonary fibrosis, by mediating myofibroblast activation, proliferation, and ECM protein deposition [14,15,16,17].

#### 2.1.4. Gene Mutation

Cystic fibrosis (CF) is a monogenetic disease induced by genovariation in the cystic fibrosis transmembrane conductance regulator (CFTR). Mutation in the CFTR gene affects the rheology of CF mucus, which impairs the mucociliary clearance of the respiratory tract. CF is related to the interaction of multiple intrinsic and extrinsic factors, such as genotype, mucus composition and motility abnormalities, chronic inflammation, and chronic airway infections [18].

#### 2.1.5. Idiopathic Pulmonary Fibrosis

IPF is the consequence of reiterative epithelial cell injury and severe abnormal wound healing, and it is characterized by AEC injury, activation and proliferation of fibroblasts, and accumulation of ECM. The progression of IPF is influenced by many factors, including genetic predisposition, aging, and environmental factors. However, the magnitude of the contribution and the sequence of the pathogenic causes are indeterminate [19].

### 2.2. Source of Myofibroblasts in Fibrotic Lung Tissues

**Figure 2:**
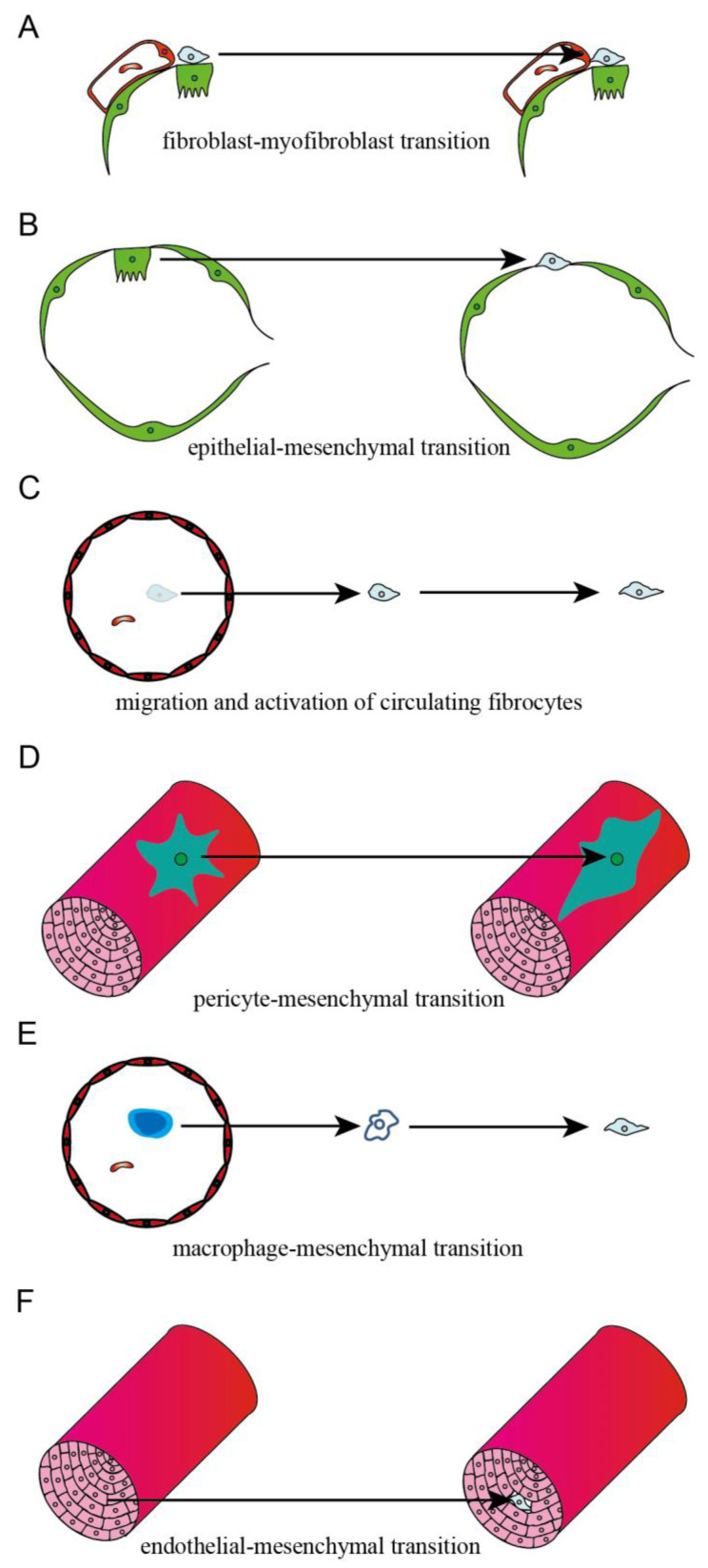
Source of myofibroblasts in fibrotic lung tissues.

Studies have shown that myofibroblasts in fibrotic lung tissue exhibit a high heterogeneity. Early studies have found that myofibroblasts were mainly derived from interstitial fibroblasts, epithelial cells, and fibroblasts in peripheral blood circulation. Recent studies indicated that endothelial cells, microvascular pericytes, and macrophages could also transform into myofibroblasts in fibrotic lung tissue (Figure 2).

#### 2.2.1. Interstitial Fibroblast

The damage of lung tissue in the process of pulmonary fibrosis induces the infiltration of immune cells, and then TGF-β derives from immune cells leads to the activation and differentiation of lung fibroblasts into myofibroblasts. In addition, TGF-β activates the cytoplasmic SMAD-2/3 complexes and stimulates the nuclear transportation of SMAD-2/3/4 complexes, which binds to the binding elements in the promoters of α-SMA, collagen, and other related genes, and leads to the synthesis and remodeling of ECM [20] (Figure 2A).

#### 2.2.2. Epithelial Cell

Epithelial and mesenchymal cell markers were co-located in IPF lung tissue; studies have indicated that TGF-β was a primary contributor of EMT. TGF-β decreased the expression of epithelial cell marker gene E-cadherin through transcription factors, including Snail, Slug, Twist, Zeb1, SIP1, and E12, while it induced the expression of mesenchymal cell marker gene N-cadherin, vimentin, and α-SMA [21]. Rock JR et al. used the SFTPC-CreERT2 knock-in allele to track the fate of AECII in vivo and found that mesenchymal cell marker genes in epithelial cells do not upregulate after the BLM challenge, and labeled epithelial cells have not been found to transdifferentiate into a myofibroblast [22]. It was suggested that there were less epithelial-derived fibroblasts in animal models of PF or that AECII cells are not major contributors to myofibroblasts in PF (Figure 2B).

#### 2.2.3. Circulating Fibrocyte

Fibrocytes are bone marrow-derived leukocytes, which infiltrate into lung tissues with the induction of CXCL12 and differentiate into fibroblasts or myofibroblasts, leading to the excessive accumulation of ECM during PF [23]. A platelet-derived growth factor (PDGF), such as Cxcl12, is a powerful chemoattractant of fibroblasts and directly contributes to fibrocyte infiltration into damaged lungs. Imatinib (PDGFR-blocking antibody) significantly reduces fibrocyte migration in vitro and decreases the number of fibrocytes in the lungs after a BLM challenge [24]. These studies demonstrated that circulating fibrocytes presented a crucial role in the development of PF by transdifferentiating into a myofibroblast (Figure 2C).

#### 2.2.4. Microvascular Pericyte

Pericytes are mesenchymal cells located at the abluminal surface of the endothelium of microvessels and help to keep the homeostasis of microvascular and participate in angiogenesis [25]. Sava et al. reported that the pericytes marker NG2 co-localized with α-SMA in IPF, and α-SMA^+^ pericytes accumulated with the treatment of IPF lung ECM [26]. Wang et al. reported that miR-107 was significantly decreased in ECs of fibrotic lung tissue, which induced the activation of the HIF-1α/Notch1/PDGFRβ/YAP1/Twist1 axis and promoted the expression of α-SMA and Coll1A, thus contributing to the fibrotic transdifferentiation of pericytes [27] (Figure 2D).

#### 2.2.5. Macrophage

The macrophage–myofibroblast transition (MMT), which is found in kidney fibrosis, is a recent term. It indicates that macrophages, derived from circulating monocytes originating in the bone marrow, could transform into myofibroblasts and contribute to kidney fibrosis [28]. Yang et al. found that most of the myofibroblasts in PF were derived from macrophages; in addition, the majority of MMT cells in the fibrotic lungs presented the M2 phenotype [29] (Figure 2E).

#### 2.2.6. Endothelial Cell

Hashimoto et al. established BLM-induced lung fibrosis in Tie2-Cre/CAG-CAT-LacZ mice, in which LacZ was stably expressed in pan-ECs, and they found that X-gal-positive cells were presented in a lung fibroblast from BLM-challenged mice. This indicated that lung capillary ECs could be active and transdifferentiate to fibroblasts via the E(nd)MT in BLM-challenged mice. Furthermore, 16% of fibroblasts were derived from the ECs in BLM-induced PF [30]. Arterial layers’ structural changes and the deposition of collagen and elastin were presented at the adventitia of IPF patients. Compared to NC, the expression of mesenchymal biomarkers *N*-cadherin, S100A4, and vimentin was significantly increased in the arterial layers of IPF patients, indicating that resident ECs could transdifferentiate to mesenchymal cells in PF [31] (Figure 2F).

## 3. Endothelial–Mesenchymal Transition

### 3.1. Endothelia Cell

The vascular EC monolayer is formed by tightly adjoining and connecting ECs. EC monolayers are mainly responsible for vascular permeability and the exchange between circulating blood and tissue fluids [32]. Studies indicated that ECs exhibit high heterogeneity in organs and vascular bundles, and show different roles in physiological and pathological conditions [33].

### 3.2. Injury and Activation of Endothelial Cells

Adult ECs remain quiescent in a healthy state but can be rapidly activated after injury or during pathological conditions. BLM damaged the alveolar morphology integrity, and large areas with densely packed vessel bulks were observed in the fibrotic foci in IPF. The vessels were arranged in a chaotic tumor vascular pattern with numerous tortuous and blind-ending vessels [34]. PF induced by various pathogenic factors leads to the injury of AECs and capillary ECs and results in the recruitment of immune cells by releasing the inflammatory factors [35]. In fact, various forms of EC activation would lead to the expression of “mesenchymal” genes. With the persistent activation of ECs, they may develop into endothelial dysfunction and eventually into a comprehensive cell fate change, namely E(nd)MT. Repetitive lung injury induced the activation of pulmonary capillary endothelial cells (PCECs) and perivascular macrophages, thereby hindering alveolar repair and promoting fibrosis. During this process, CXCR7 expression was inhibited in pulmonary capillary endothelial cells (PCECs) and VEGFR1^+^ macrophages were recruited to the perivascular area, which was regulated by Notch ligand Jagged1 in PCECs stimulated by Wnt/β-catenin, thus stimulating Notch signaling in perivascular fibroblasts and enhancing fibrosis [36]. Liu et al. demonstrated that (Salvianolic acid B) SAB inhibited PF by protecting ECs against oxidative stress [37]. These studies indicated that ECs were injured in the initial stage of PF and aggravated the progress of PF.

### 3.3. Endothelial–Mesenchymal Transition

The microstructure shows that ECs in the fibrotic area of PF lose their endothelial characteristics, such as polarity and intercellular junctions, while acquiring mesenchymal cell characteristics, such as a spindle-shaped cell morphology and increased migration ability [38]. In addition, the results of immunofluorescence staining and single-cell sequencing also showed that the expression of mesenchymal cell marker proteins was also observed in these ECs [39,40]. It has been reported that vascular damage occurred first in radiation-induced lung fibrosis (RIPF), including narrowing and obliterating capillaries induced by EC swelling, fibrin deposition, and endothelial hyperplasia [41]. The E(nd)MT appeared before EMT in RIPF in mouse lung; in addition, radiation-induced hypoxia mediated activation of TGFβ receptor I/Smad signaling via HIF1α, thus promoting E(nd)MT [42]. In systemic endotoxemic-induced PF, the expression of DPP-4 was significantly increased in PVECs in both the presence and absence of immune cells. DPP-4 inhibitors decreased ROS production and E(nd)MT in LPS-induced lung injury [43]. These studies showed that E(nd)MT was evident in both pulmonary fibrosis and lung injury. E(nd)MT leads to disruption of the vascular barrier, and the changes in the endothelial cell phenotype and function can further induce immune infiltration. In the BLM-induced mouse PF, macrophages, especially M2 macrophages, were significantly increased in the lung tissue. M2, but not M1 that was isolated from mouse lung tissue, promoted the fibroblasts–myofibroblasts transition through Wnt/β-catenin. During the alveolar inflammation phase, M1 macrophages infiltrated into lung tissue and secreted MMP2 and MMP9, which promoted the degradation of ECM and disruption of the vascular barrier and resulted in the infiltration of more inflammatory cells into the injured tissue [44]. Above all, E(nd)MT leads to the increase in vascular barrier permeability and immune cell infiltration into injured lung tissues. Immune cells promoted the degradation of ECM and destruction of the vascular barrier by secreting various cytokines, which in turn promote PF by activating fibroblasts.

### 3.4. Inducement of E(nd)MT

Under pathological conditions, many factors such as hypoxia, oxidative stress, inflammatory factors, and disturbed shear stress can induce E(nd)MT.

#### 3.4.1. Hypoxia

The patient’s lung function deteriorates rapidly as the PF progresses, and severe hypoxia occurs even at rest in the final stages. Hypoxia is associated with the increase in macrophage accumulation and activation, oxidative stress, and profibrogenic/pro-angiogenic cytokine activity which contributes to pulmonary injury [45]. An in vivo study showed that the lung vascular system of hypobaric hypoxic rats showed obvious structural remodeling with elevated perivascular collagen deposition, increased wall thickness, and reduced lumen diameter. An in vitro study showed that the expression of HIF1α and α-SMA in human pulmonary microvascular endothelial cells (HPMECs) was increased in hypoxia, while that ofCD31 was decreased. In addition, phosphorylation of Smad3 and the expression of TGFβ receptor I and Snail1 were significantly increased. This indicated that the hypoxia-induced E(nd)MT was consistent with HIF1α [42].

#### 3.4.2. Oxidative Stress

BLM is commonly used in rodent models of PF. This binds to metal ions chelation and induces the formation of pseudoenzymes, and then results in the generation of superoxide and hydroxyl radicals, thus targeting DNA strand breaks [46]. An increase in ROS in endothelial cells and an impaired endothelial barrier were also found in both LPS-induced acute lung injury and LPS-treated ECs in vitro [47]. ROS caused endothelial DNA stress and initiated repair processes, including Wnt signaling [48]. BLM induces endothelial stress and destroys Wnt balance, thus causing the activation of ECs, which may ultimately lead to PF [49].

#### 3.4.3. Inflammatory

Inflammation creates an ideal environment for the development of E(nd)MT. E(nd)MT is aggravated under the inflammatory microenvironment [50]. Inflammatory factors, including IL-1β, TNF-α, endotoxin, etc., are upregulated in lung tissue in PF and activate ECs and induce E(nd)MT [51,52]. This transition occurs during the initial stages of tissue remodeling, which is critical for the development of fibrosis. Donato et al. firstly proved that the impaired vascular endothelial function of healthy older humans was associated with nuclear transportation of NFκB in ECs, low IκB expression, and increased expression of IL-6, TNF-α, and MCP-1 [53]. This is consistent with the finding that ECs dysfunction and fibrosis are persistent in elderly men [54,55].

#### 3.4.4. Shear Stress

Pulmonary hypertension (PH) is a common complication of ILD, including IPF, with a reported occurrence from 32 to 85%. The cross-section of the pulmonary vascular system becomes smaller in the areas with severe PF, which leads to the increase in pulmonary vascular resistance, and vascular ablation may also lead to increasing pulmonary vascular resistance [56]. ECs sense flow and transmit mechanical signals through mechanosensitive signaling pathways and activate molecules that induce phenotypic and functional changes [57]. Krüppel-like factor 2 (KLF2), a shear-responsive transcription factor, is a critical regulator of endothelial gene expression patterns induced by an atheroprotective flow and serves as a “molecular switch” in regulating endothelial function. KLF2 has been reported to promote angiogenesis and reduce E(nd)MT [58]; however, it was downregulated in BLM-induced rat pulmonary fibrosis [56]. This indicated that the decrease in KLF2 in IPF ECs promoted the activation of ECs and E(nd)MT.

### 3.5. Signaling Pathways That Regulate Endothelial–Mesenchymal Transition

**Figure 3:**
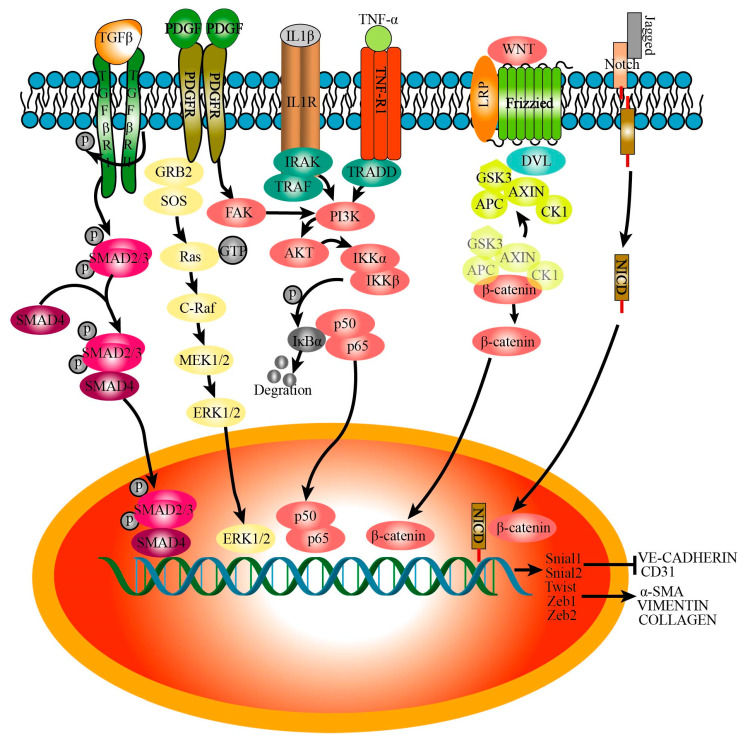
Signaling pathways that regulate endothelial–mesenchymal transition.

Epithelial cells, ECs, and immune cells that infiltrate into lung tissue secrete various cytokines after lung injury, which lead to the injury, activation, and E(nd)MT of endothelial cells. The E(nd)MT is mainly regulated by the following signals (Figure 3).

#### 3.5.1. TGFβ

TGF-β, as the strongest profibrotic mediator, leads to recruitment and activation of monocytes, circulating fibrocytes, and fibroblasts, and it can be released by alveolar epithelial cells, ECs, macrophages, platelet granules, and infiltrating regulatory T cells [59]. ECs are also activated and increase the expression of TGF-β and TGFβR1 in BLM-induced mice PF. Initially, TGF-β binds to the type II receptor (TGFβRII); however, TGFβ has been shown to possess a higher affinity for TGFβRI than TGFβRII, and upregulation of TGFβRI in ECs induced the phosphorylation of Smad2/Smad3, which then formed heteromeric complexes with SMAD4. In the nucleus, with the help of transcriptional co-activators or co-repressors and chromatin remodelers, Smad complexes can bind to the promoter regions of target genes, such as Snai1, Snai2, Twist, Zeb1, and Zeb2, thus promoting the expression of α-SMA and collagen I while decreasing the expression of VE-cadherin and CD31 [60,61,62].

#### 3.5.2. IL-1β

The expression of IL-1β was enhanced in IPF lung tissues and BLM-induced fibrotic mice lung tissues compared to the control groups [63]. A study has found that IL-1β induced the alteration of the cellular morphology of HUVECs, from a cobblestone-like shape to a spindle-like shape. In addition, IL-1β activated Smad via BMP2 and BMP7 rather than BMP4 [64]. IL-1β also increased IRAK and TRAF6 expression and led to the activation of PI3-kinase, and then induced the phosphorylation of IKKα/β and degradation of IκB, thus activating NF-κB [65], which induced E(nd)MT [66].

#### 3.5.3. TNFα

TNFα could induce the activation of fibroblasts and promote collagen production and has been proved to be significantly increased in the lungs of IPF patients [67]. TNFα binds to TNFR1 and activates TRADD, and then promotes the phosphorylation and nuclear translocation of NFκB through the downstream of PI3K/AKT [68]. NFκB can regulate the expression of transcription factors Snail and Zeb [69], which inhibit the expression of CD31 and promote the expression of COL1A1 and E(nd)MT.

#### 3.5.4. Wnt

Wnt signaling is well-known for its role in developmental biology, including cardiovascular development, and plays a crucial role in vascular and cardiac disease [70]. Wnt/β-catenin has been reported to regulate the differentiation of lung epithelial cells and fibroblasts, and the pathogenesis of PF [71]. The Wnt ligand binds to membrane receptor complex Frizzled and the coreceptor LRP5/6; activates DVL, which promotes the aggregation of the AXIN, APC, CK1α, and GSK3β complexes to the receptor; promotes the phosphorylation of GSK3β; and induces the accumulation and nuclear translocation of β-catenin [70]. Then, β-catenin regulates the expression of ECs and mesenchymal cell markers and E(nd)MT through Snail [72].

#### 3.5.5. Notch

The excessive secretion of cytokines in ECs of BLM-induced fibrotic rat lung tissue and multiple BLM-induced fibrosis mouse lung tissue promotes the expression of Jagged1 and activates the Notch1 signaling pathway in PMECs [36,73]. In addition, Liebner et al. discovered that Jagged/Notch promoted E(nd)MT through β-catenin-induced Snail transcription during cardiac pad development [74].

#### 3.5.6. PDGF

In BLM-induced PF, PDGF released by alveolar macrophages induces the loss of intercellular adhesion and the alteration in the cellular morphology of ECs [75]. PDGF leads to the activation of small GTPaseRas/ERK, phosphatidylinositol 3’-kinase/Akt, and FAK [76], and then PI3K/AKT signaling affects the expression of endothelial markers such as vWF and CD31 through transcription factor Twist1 [77]. Hypoxia or monocrotaline (MCT) induced the expression of PDGF-B and TGF-β1 as well as a positive-feedback loop between them; hypoxia or MCT induced the E(nd)MT of PAECs by NEP/PDGF-B/TGF-β1. Moreover, E(nd)MT may be mediated by Snail, Zebs, and Twist [78].

#### 3.5.7. Others

FGF played an important role in keeping the homeostasis of ECs; however, it presented an unexpected role in E(nd)MT during the pathological condition [79]. In addition, FGF2 increased the expression of miR-20a and inhibited TGFβ1-induced E(nd)MT via suppressing receptor complex levels and activating Smad2 and Smad3 [80]. However, Lee et al. indicated that IL-1β combined to the canonical pathway and PI3-kinase and led to the upregulation of FGF-2 through NF-κB, thus mediating E(nd)MT [65]. The existing studies on the FGF regulating E(nd)MT remain controversial, which may be caused by the heterogeneity of ECs. A preclinical study of PF showed that the expression of VEGF was significantly decreased in fibrotic lesions of rats but increased in epithelial cells and ECs [81]. VEGF played a vital role in regulating angiogenesis and vascular permeability and was significantly upregulated in the serum of cystic fibrosis (CF). VEGF inhibited TGF-β and SMAD2 and the expression of collagen via VEGF-R2, thus inhibiting E(nd)MT [82]. Integrin β1 induces the activation of ECs associated with VEGF-R1, which could bind to VEGF-A and lead to a decrease in the amount of VEGF-A that interacts with VEGF-R2 and induces E(nd)MT [83]. A study has indicated that the actions of Ponatinib (AP) reduced E(nd)MT through decreasing the expression of FGF-2, VEGF, and vimentin, whereas AP induced the expression of VE-cadherin [84]. These studies indicated that the role of FGF-2 and VEGF in E(nd)MT was complex.

## 4. Effects of Activated Endothelial Cells on other Cells in Pulmonary Fibrosis

### 4.1. Recruitment of Immune Cells

ECs play an important role in both innate and adaptive immune responses, and they are known as “conditional innate immune cells” and perform an important role in various inflammatory and immune pathologies [85]. In BLM-induced PF, the expression of many adhesion molecules (E-selectin, P-selectin, and CD34) and chemokines (CXCL1, CXCL2, CCL2, CCL3, CCL6, CCL7, and CCL9) was significantly increased in ECs. This suggested that ECs were activated in PF and contributed to the infiltration of immune cells into the injured lungs [86]. A study has reported that injured ECs induced by MMP19 promoted the recruitment of immune cells by the SDF1/CXCR4 axis in vitro and in vivo [87]. In mouse atherosclerosis, CXCR4 expressed by damaged ECs promoted the recruitment of monocytes to the damaged site, especially in the plaque area [88]. Cao et al. reported that repeated BLM or hydrochloric acid treatment led to the activation of vascular ECs, and then the activated ECs recruited VEGFR^+^ macrophages to the vicinity of damaged blood vessels, and the Wnt signal secreted by VEGFR^+^ macrophages promoted the expression of Jag1 in ECs, which in turn affects the activation of perivascular fibroblasts [36]. Thus, damaged ECs in PF promote the recruitment of immune cells by chemokines, which in turn activate the ECs.

### 4.2. Activation and Myofibroblastic Transformation of Fibroblasts

Activated ECs induce the secretion of many cytokines and profibrotic mediators, including TGF-β, CTGF/CCN2, and PAI-1, which directly contribute to the recruitment and activation of fibroblasts to produce collagen [86]. Irradiation treatment promotes the expression of Snail in human umbilical vein endothelial cells and induces E(nd)MT. After co-culturing with the endothelial cells, the expression of α-SMA in MRC-5 was significantly upregulated, which indicated that irradiated ECs that underwent E(nd)MT promoted fibroblast differentiation into myofibroblasts [89]. Qian et al. found that the expression level of collagen I in fibroblasts was significantly increased after co-culturing with ECs that were isolated from mouse lung tissue treated with BLM; in addition, activated ECs in BLM-treated mouse lung tissue resulted in an increased secretion of TGF-β1 and CTGF, which in turn promoted the activation, collagen synthesis, and transformation of resident fibroblasts [90].

### 4.3. Epithelial Cell Damage and Repair

The air–blood barrier mainly includes the liquid layer containing pulmonary surfactant, the alveolar epithelial cell layer, the epithelial basement membrane, the space between the alveolar epithelium and the capillaries, the basement membrane of the capillaries, and the capillary EC layer [91]. ECs and epithelial cells are close in space, so activated ECs must influence the physiological activity of the epithelial cells. A study has demonstrated that the loss of epithelial barrier function in acute lung injury was induced by endothelial activation and injury rather than direct epithelial damage [92]. Chio et al. showed that E(nd)MT occurred before EMT in an irradiation-induced mouse model of pPF, and 2-ME, a metabolite of 17-β-estradiol, could inhibit the irradiation-induced HIF-1α in ECs, which in turn inhibited E(nd)MT. Interestingly, they also found that EMT was inhibited in 2-ME-treated mice, suggesting that EC activation promoted epithelial cell damage [42]. Cao et al. reported that overexpression of CXCR7 in PCECs inhibited the damage of epithelial cells and PF after a single round of administration of BLM or hydrochloric acid, while repeated administration inhibited the expression of CXCR7 and induced the recruitment of VEGFR1^+^ perivascular macrophages. This recruitment stimulates upregulation of the Jagged1 in PCECs, thus stimulating and enhancing the damage of epithelial cells and PF [36]. Jiao et al. found that 27-Hydroxycholesterol (27HC) induced E(nd)MT in vascular ECs, and the process promoted EMT and migration in breast cancer cells through the alteration of the tumor microenvironment [93]. This was consistent with the studies that VEGF and syndecan-1 in the tumor stroma were significantly upregulated in dysfunctional ECs, simulated angiogenesis, and induced the migration and EMT of epithelial cells [94,95].

### 4.4. Pericytes Cell Activation

It has been well-known that the crosstalk between ECs and pericytes influences not only normal physiology but also disease development [96]. In BLM-induced PF, oxidative stress induces DNA stress of endothelial cells and breaks the Wnt balance of ECs, which promotes the activation and phenotypic change in pericytes [48]. In addition, the expression of microRNA let-7d in exosomes from pulmonary vascular endothelial cells was significantly suppressed, which induced the expression of TGFβRI in pericytes, and promoted the nuclear transportation of Smad3 and stabilization of the Smad3/4 protein complex, thus increasing the expression of fibrotic genes [97].

**Figure 4:**
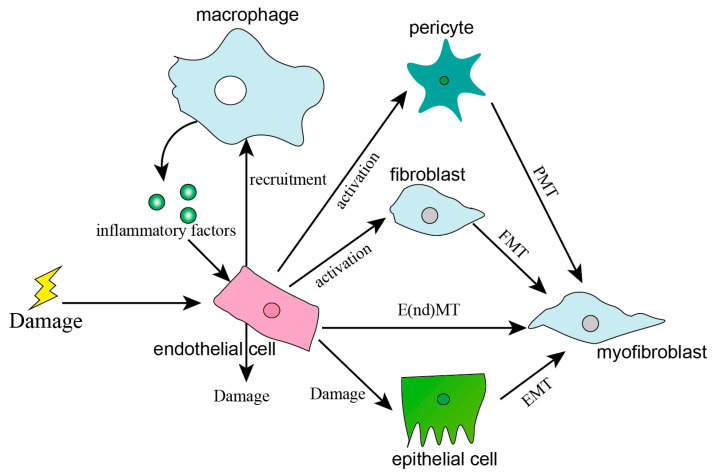
Crosstalk between endothelial cells and other cells in pulmonary fibrosis.

In summary, the adhesion molecules and chemokines expressed by activated ECs in PF recruited immune cells to the injured blood vessels, and then cytokines secreted by immune cells promoted ECs injury, which in turn inhibits the proliferation and repair of epithelial cells, while promoting the activation and proliferation of fibroblasts and pericytes, and ultimately promoting PF (Figure 4).

## 5. Drugs for IPF That Targeted Endothelial Cells

### 5.1. Ambrisentan, Bosentan, and Macitentan

Endothelin 1 (ET-1) is involved in the pathogenesis of endothelial dysfunction and PF in the rodent PF model, and inhibition of its receptors can retard PF [98]. Ambrisentan, bosentan, and macitentan were endothelin receptor antagonists; all of them could inhibit endothelial cell injury and impair endothelial function. Ambrisentan, an ETA receptor–selective antagonist, could inhibit ET-1-induced E(nd)MT and fibroblast activation. A clinical trial of ambrisentan showed that there was no difference in FVC, DLCO, and 6MWD between patients treated with a placebo and those treated with ambrisentan at week 48 [99]. Bosentan was an oral antagonist of ETA and the ETB receptor; however, research has indicated that the expected objective of bosentan in an ILD-3 trial did not appear, and there were no effects of bosentan on health-related quality of life or dyspnea [100]. From a phase II trial of the endothelin receptor antagonist macitentan, it was reported that there were no differences observed between the treatment and placebo in pulmonary function tests or time to disease worsening or death [101].

### 5.2. Rapamycin

PI3Ks and mTOR are crucially important to the pathogenesis of IPF. Omipalisib, a PI3K/mTOR inhibitor, has been used in a clinical trial study of IPF, and the results indicated that PI3K-targeted engagement had a demonstrable effect on lung metabolism in fibrotic lung. This suggested that the inhibition of PI3K can be a feasible strategy to the remedy of IPF; however, later phase trials would be urgently needed to evaluate its function on fibrosis [102].

### 5.3. Imatinib

Imatinib, a tyrosine kinase inhibitor (TKI), can inhibit the activity of PDGF receptors (PDGFR-a and -b), whereas, compared with the placebo-controlled group, imatinib did not affect survival or lung function of IPF patients followed for 96 weeks [103].

### 5.4. Relaxin

Relaxin, an inhibitor of the Notch pathway, was used for treating scleroderma. Interestingly, researchers indicated a tendency for slight improvements in skin thickening in patients who received the placebo, as well as a tendency for the slow loss of pulmonary function [104].

### 5.5. Nintedanib

Nintedanib, a tyrosine kinase receptor inhibitor, inhibits the activation of the PDGF receptor, FGF receptor, and VEGF receptor, and it is one of the first antifibrotic drugs to be approved for use in IPF. In a clinical trial, researchers have reported that nintedanib significantly recovers the forced vital capacity (FVC) of IPF patients over the 52-week treatment [105]. In vitro studies have indicated that nintedanib interferes with the proliferation, migration and differentiation, and secretion of ECM of fibroblasts [106]. Nintedanib has also been reported to inhibit E(nd)MT via targeting the VEGF/FAK signaling pathway [107]. In pulmonary arteries (PAs), nintedanib attenuated the expression of mesenchymal markers of HPMVECs and HPASMCs, inhibited the phosphorylation of PDGF and FGF receptors, and reduced neointimal lesions and medial wall thickening in PAs. Additionally, the expression of Twist1 in lung tissue was significantly reduced by nintedanib [75]. The research also reported that the circulating endothelial cells in IPF patients treated with nintedanib was higher than the control group, which further indicated that nintedanib affects the blood vessels in IPF [108].

### 5.6. Pirfenidone

A clinical analysis by Traci N. Adams showed that the FVC of the pirfenidone group was dramatically recovered compared to the placebo group [109]. Pirfenidone inhibits the activation of the TGF-β1/mTOR/p70S6K signaling pathway, thus reducing the proliferation of intestinal fibroblasts and the expression of collagen I [110]. Pirfenidone has also been reported to reduce pulmonary fibrosis in silicosis rats by inhibiting macrophage polarization via the JAK2/STAT3 signaling pathway [111]. Therefore, pirfenidone could inhibit fibrosis and inflammation, and it has been used for controlling IPF. Zhang et al. reported that pirfenidone decreased pulmonary fibrosis by inhibiting E(nd)MT and reduced the expression of Gli1 and α-SMA in LPS-induced ARDS [112]. Pirfenidone treatment also decreased the radiation-induced expression of TGF-β1 and phosphorylation of Smad3 in AECs and ECs [113]. This indicated that pirfenidone may affect the blood vessels in PF.

## 6. Conclusions

ECs play an important role in PF. As an important source of myofibroblasts in PF, activated ECs transdifferentiate into myofibroblasts and promote the proliferation of ECCs-derived myofibroblasts. On the other hand, activated ECs promote the recruitment and infiltration of immune cells. Finally, activated ECs also secrete cytokines to affect the physiological activities of neighboring epithelial cells and pericytes cells, thus promoting PF. E(nd)MT occurs at the beginning stage of PF and has been demonstrated to aggravate PF. Many drugs for IPF that target ECs have been applied in the clinical trial studies; however, there was no effective drug for IPF exploited until now. This may be due to E(nd)MT being a complex progress and regulated by various factors, and we still know relatively little about the molecular mechanism in E(nd)MT. The mechanism of the crosstalk between ECs and other cells is another important area ripe for future investigation. However, the tight regulation of ECs activation and E(nd)MT could be promising targets for manipulation in PF.

## Data Availability

The datasets presented in this study can be found in online repositories. The names of the repository/repositories and accession number(s) can be found in the article.

## List of Abbreviations

PF	Pulmonary fibrosis
ECM	Extracellular matrix
E(nd)MT	Endothelial–mesenchymal transition
EMT	Epithelial–mesenchymal transition
ECs	Endothelial cells
AECII	Type II alveolar epithelial cells
MMP	Matrix metalloproteinases
BLM	Bleomycin
IPF	Idiopathic pulmonary fibrosis
MMT	Macrophage–mesenchymal transition
FMT	Fibroblast–mesenchymal transition
